# ‘High hopes for treatment’: Australian stakeholder perspectives of the clinical translation of advanced neurotherapeutics for rare neurological diseases

**DOI:** 10.1111/hex.14063

**Published:** 2024-05-06

**Authors:** Christina Q. Nguyen, Didu S. T. Kariyawasam, Tsz Shun Jason Ngai, James Nguyen, Kristine Alba‐Concepcion, Sarah E. Grattan, Elizabeth E. Palmer, Kate Hetherington, Claire E. Wakefield, Russell C. Dale, Sue Woolfenden, Shekeeb Mohammad, Michelle A. Farrar

**Affiliations:** ^1^ Discipline of Paediatrics and Child Health University of New South Wales Medicine and Health Sydney New South Wales Australia; ^2^ Department of Neurology Sydney Children's Hospital Network Sydney New South Wales Australia; ^3^ Centre for Clinical Genetics Sydney Children's Hospital Network Sydney New South Wales Australia; ^4^ Behavioural Science Unit Kids Cancer Centre, Sydney Children's Hospital Sydney New South Wales Australia; ^5^ Children's Hospital Westmead Clinical School Faculty of Medicine and Health, Sydney Medical School University of Sydney Sydney Australia; ^6^ Population Child Health Research Group University of New South Wales Sydney New South Wales Australia; ^7^ Sydney Institute for Women, Children and their Families Sydney Local Health District Sydney New South Wales Australia

**Keywords:** advanced therapeutics, clinical decision‐making, paediatric neurology, patient advocacy, precision medicine, rare diseases

## Abstract

**Introduction:**

Advanced therapies offer unprecedented opportunities for treating rare neurological disorders (RNDs) in children. However, health literacy, perceptions and understanding of novel therapies need elucidation across the RND community. This study explored healthcare professionals' and carers' perspectives of advanced therapies in childhood‐onset RNDs.

**Methods:**

In this mixed‐methodology cross‐sectional study, 20 healthcare professionals (clinicians, genetic counsellors and scientists) and 20 carers completed qualitative semistructured interviews and custom‐designed surveys. Carers undertook validated psychosocial questionnaires. Thematic and quantitative data analysis followed.

**Results:**

Participants described high positive interest in advanced therapies, but low knowledge of, and access to, reliable information. The substantial ‘therapeutic gap’ and ‘therapeutic odyssey’ common to RNDs were recognised in five key themes: (i) unmet need and urgency for access; (ii) seeking information; (iii) access, equity and sustainability; (iv) a multidisciplinary and integrated approach to care and support and (v) difficult decision‐making. Participants were motivated to intensify RND clinical trial activity and access to advanced therapies; however, concerns around informed consent, first‐in‐human trials and clinical trial procedures were evident. There was high‐risk tolerance despite substantial uncertainties and knowledge gaps. RNDs with high mortality, increased functional burdens and no alternative therapies were consistently prioritised for the development of advanced therapies. However, little consensus existed on prioritisation to treatment access.

**Conclusions:**

This study highlights the need to increase clinician and health system readiness for the clinical translation of advanced therapeutics for RNDs. Co‐development and use of educational and psychosocial resources to support clinical decision‐making, set therapeutic expectations and promotion of equitable, effective and safe delivery of advanced therapies are essential.

**Patient or Public Contribution:**

Participant insights into the psychosocial burden and information need to enhance the delivery of care in this formative study are informing ongoing partnerships with families, including co‐production and dissemination of psychoeducational resources featuring their voices hosted on the Sydney Children's Hospitals Network website SCHN Brain-Aid Resources.

## 1 INTRODUCTION

While individually uncommon, rare diseases affect approximately 1 in 12 individuals.^1^ From approximately 8000 known rare diseases, 80% are genetic in origin, 70% have a neurological component and the majority have childhood onset.^2^ Currently, less than 5% of these conditions are amenable to therapeutic modulation, such that mortality rates before the age of 5 years remain as high as 30%.^3^ Furthermore, their progressive course is associated with a high prevalence of neurodisability. Advanced therapies, defined here as gene therapy, stem cell therapy, neurostimulation and experimental neuroimmunotherapies, offer new opportunities for the treatment and amelioration of many paediatric rare neurological disorders (RNDs). Alongside the promise of great technological advancements, however, comes the need for ethically responsible research and clinical administration inclusive of the voices of people living with RNDs as valued partners.

With individualised medicines now possible, technologies are set to expand across modalities and therapeutic areas, potentially disrupting conventional therapeutic pipelines worldwide.^4^ In 2018, the first gene therapy for spinal muscular atrophy (SMA), onasemnogene abeparvovec, was approved after clinical trials demonstrated improved survival and motor outcomes in the presence of a favourable safety profile.^5^ As of 2022, this adeno‐associated virus‐mediated gene therapy has received Australian regulatory approvals and has been integrated within the public health system, which provides specialist and public hospital services and pharmaceuticals. Using the feasibility and clinical utility of gene therapy in SMA as a template condition for other RNDs, there are now over 3600 advanced therapies^6^ at various stages of development and implementation within the preclinical, clinical trial and translational pipeline, and a move by policy and practice stakeholders to accelerate, provide, deliver and harmonise therapeutic access for rare and ultra‐RNDs. These innovative technologies have created wide‐reaching and unparalleled opportunities to practise precision medicine within a proactive treatment paradigm.

Matching the expanding horizon of medical advancement, therapeutic and ethical uncertainties are emerging within the clinical domain. Unintended consequences of gene therapies, namely, death, acute liver toxicity and renal failure, temper enthusiasm, underpinning the need for education across stakeholders on the risk–benefit profiles of novel agents. While families of children with RNDs value and seek evidence‐based, current and collated educational resources at diagnosis,^7^ a recent Patient Think Tank in The Netherlands highlighted knowledge gaps connected with therapies and advised more patient education and involvement about rare disease clinical trials from an early stage.^8^ Similarly, surveys and workshops to advance treatment of rare diseases undertaken by the Global Genes Patient Identification and Engagement for RARE CNS Disorders multistakeholder initiative have recommended education and dialogue to bridge gaps.^9^ With increased activity and innovations in advanced therapies, the US Food and Drug Administration has published guidance for the development of drugs and biologics for rare diseases, which also encourages involvement of people living with rare diseases in the drug development process.^10^

As the first Australian study to comprehensively assess both perspectives and information needs of health professionals and carers of children with RNDs, we hypothesise that stakeholders will have a high interest in the role and potential of advanced therapeutics; however, information needs are mismatched with the availability of high‐quality educational resources that directly meet the needs and priorities of stakeholders. Accordingly, this study aims to investigate the perspectives, experiences and expectations of advanced therapies in Australian healthcare professionals working in the field of RNDs and carers of affected children. It also aimed to identify the concerns, strategies and information and support needs surrounding treatment delivery and decision‐making.

## 2 METHODS

A convergent parallel mixed‐methods design, where qualitative and quantitative data were collected concurrently, was used for this formative study. This approach was utilised to provide further depth and understanding of perspectives, comparison and validation of one set of results with the other and to measure coping skills, social supports and well‐being of carers to inform future health practices.

### 2.1 Participants and recruitment

To ascertain a range of experiences from as many different perspectives as possible, purposive sampling was used. Consistent with this approach, the varied characteristics sought amongst health professionals included age, gender, geographical location in Australia and profession connected to RNDs (neurologist, geneticist, scientist and allied health). Similarly, amongst carers, efforts were made to ensure diversity in the RND conditions, experiences of accessing advanced therapeutics and to include rural/remote regions. Primary carers of children with RNDs who have considered, undertaken or are currently undergoing advanced therapies were identified through the Sydney Children's Hospitals Network (SCHN) neurology and clinical genetics clinics and invited to participate. Recruitment occurred between April 2021 and July 2021, continuing until sampling requirements for study design, informational saturation and diversity in participant characteristics and treatment choices were met. Individuals who were unable to speak conversational English unaided by a translator were excluded. The study was approved by the SCHN Human Research Ethics Committee (2020/ETH02748). Potential participants were contacted via email with an invitation letter and followed up with a subsequent email reminder 2 weeks later. Written informed consent was obtained from all participants.

### 2.2 Data collection

Semistructured interviews were conducted via Zoom (Zoom Video Communications Inc.) and involved one participant, an interviewer and a facilitator. The facilitator served as an independent observer, who ensured that the interviewer maintained objectivity and served as an information source around the project. An interview guide was used to discuss participant experiences, perspectives and expectations of treating RNDs (Supporting Information S1: Material 1). While interview guides were used to discuss participant experiences, perspectives and expectations of treating RNDs, the phrasing and sequencing of questions were open and flexible to support wider discussion. This was to give participants a level of autonomy during the conduct of the study, while also keeping a consistent set of topics across individuals. Member‐checking, whereby participants validated the accuracy of investigator interpretations, was used, whereby participants validated the accuracy of investigator interpretations.

Following interviews, participants were emailed an invitation to complete an online questionnaire (Qualtrics XM). Questionnaires for professionals and carers were co‐developed by a multidisciplinary team (neurologist, psychologist, developmental paediatrician and research associate) and pilot‐tested to ensure that all questions were appropriate and understood. Questionnaires incorporated 5‐point Likert scales and short answer responses to assess attributes, strategies, information and supports for guiding the successful implementation of these therapies into clinical practice from professional and carer perspectives. For carers, emotional distress, coping strategies and social supports were assessed using the following tools (Supporting Information S1: Material 2).

1. The Depression Anxiety Stress Scale (DASS‐21).^11^
2. The Coping Orientation to Problems Experienced (COPE) Inventory.^12^
3. The Multidimensional Scale of Perceived Social Support (MSPS).^13^

A psychological distress plan was in place for participants who experienced distress during or because of the interview, or if they scored ‘severely’ or ‘extremely severely’ in the DASS‐21.

### 2.3 Data analysis

The qualitative and quantitative data sets were collected and analysed separately but concurrently using a convergent parallel design. During interpretation, quantitative data were linked with the qualitative themes (C. Q. N., D. S. T. K., K. A.‐C., S. E. G. and M. A. F.). Descriptive statistics including frequencies, percentages, means, medians and range were used to explore the sample sociodemographic, role characteristics (for professionals) and psychometric scales (for carers).

The conceptual framework of Miles et al. was used to thematically analyse the interview transcripts.^14^ First, transcripts were read and annotated for salient content amongst eight researchers, with at least two researchers reading each transcript. Researchers then collaboratively established a set of overall themes and developed a coding tree. Guest et al.'s approach to assessing thematic saturation was incorporated, with the iterative process repeated until all transcripts had been interpreted.^15^ Two researchers independently coded all interview transcripts in NVivo 12 Pro, organising quotes into the previously agreed themes. This coding was compared and demonstrated a high level (95%) of concordance. Illustrative quotes were used to highlight concepts emerging from the themes. Quantitative data were analysed using descriptive statistics and graphs in IBM SPSS Statistics v26. Frequencies and percentages were reported for categorical variables.

## 3 RESULTS

### 3.1 Demographics and participant characteristics

A total of 34 primary carers and 43 professionals engaged in the care of children with RNDs were invited to participate. Twenty individuals from each group formed the study population, representing a response rate of 58% and 46%, respectively. The primary carers included 16/20 (80%) mothers, 3/20 (15%) fathers, including one parental dyad, and 1/20 (5%) female foster parent (Table 1, Supporting Information S1: Material 3). Interviews lasted (mean 47, range 31–60) minutes. Postinterview, 17/20 (85%) clinicians/scientists and 17/20 (85%) primary carers completed the custom‐designed questionnaire. 15/20 (75%) carers completed the DASS‐21, COPE and MSPS surveys. The heterogeneity with regard to professionals' specialisation, age and gender, carers' previous advanced therapeutic access and the diagnostic spectrum represented by the study sample are summarised in (Table 1).

**Table 1:** Participant demographics.

Participant demographics	Clinicians/scientists, *N* (%)			Carer, *N* (%)
Gender				
Male	8 (40%)			3 (15%)
Female	12 (60%)			17 (85%)
Age range (years)				
30–40	2 (10%)			5 (25%)
40–50	8 (40%)			10 (50%)
50–60	7 (35%)			4 (20%)
60–70	3 (15%)			1 (5%)
Geographical location in Australia				
New South Wales	17 (85%)			18 (90%)
Victoria	2 (10%)			0 (0%)
Queensland	1 (5%)			0 (0%)
Rural Australia	0 (0%)			1 (5%)
Overseas	0 (0%)			1 (5%)
Profession				
Paediatric neurologist	9 (45%)			NA
Clinical genetics/metabolic specialist	5 (25%)			NA
Laboratory scientist	2 (10%)			NA
Genetic counsellor/allied health	4 (20%)			NA
Carer type				
Mother	Nil			16
Father	Nil			3^a^
Foster parent	Nil			1
Conditions of carers' children		Example of a qualifying diagnosis		
Neurodevelopmental	7		*SCN2A*‐related condition	
Neuromuscular	5		Batten disease	
Neurodegenerative	5		Spinal muscular atrophy	
Movement disorder	2		*ADCY5*‐related condition	

*Note*: Highest education level was available for 86 carers; university (*n* = 12), certificate/diploma (*n* = 3), Year 12 (*n* = 2) and apprenticeship (*n* = 1).

Abbreviations: F, female; M, male; N, no; NA, not applicable; Y, yes.

^a^ Inclusive of one parental dyad.

All primary carers had children whose diagnosis was associated with a genetic variant. From the carer cohort, 9/20 (45%) had a child who previously accessed advanced therapies. Of these children, several (6/9; 67%) accessed these therapies through the Australian healthcare system; however, 3/9 (33%) were treated overseas. The remaining carers were aware of their child's genetic diagnosis and were highly interested in advanced therapies, but no current therapy was available for their disease.

### 3.2 Carer emotional distress, coping strategies and social supports

Elevated levels of psychological distress were reported by 8/16 (50%) carers assessed using the DASS‐21. This involved scoring higher than normal levels in at least one domain of anxiety, stress and or depression, as specified by the cut‐off scores listed in Supporting Information S1: Material 2. Specifically, elevated levels of anxiety, stress and depression were reported in 3/16 (19%), 8/16 (50%) and 6/16 (38%) of carers, respectively. The median total scores for respondents were 1 (range 0–6) for anxiety, 8 (range 2–10) for stress and 3 (range 0–7) for depression. Severe and extremely severe scores for anxiety and stress were reported in 1/16 (6%) and 1/16 (6%) of carers, respectively. Evaluation of styles adopted by carers showed a spectrum of coping mechanisms. The COPE‐Inventory determined the dominant coping style amongst carers as ‘acceptance’, ‘planning’ and ‘positive reinterpretation and growth’, with 6/16 (38%), 5/16 (31%) and 3/16 (19%) of respondents scoring highest in these coping styles, respectively. Those who scored lower in ‘planning’ and ‘positive reinterpretation and growth’ appeared to score significantly higher than average on ‘behavioural disengagement’ and ‘mental disengagement’. These families were also those who felt that they had little to no treatment options for their child.

### 3.3 Understanding and attitudes around advanced therapies and clinical trials

**Figure 1:**
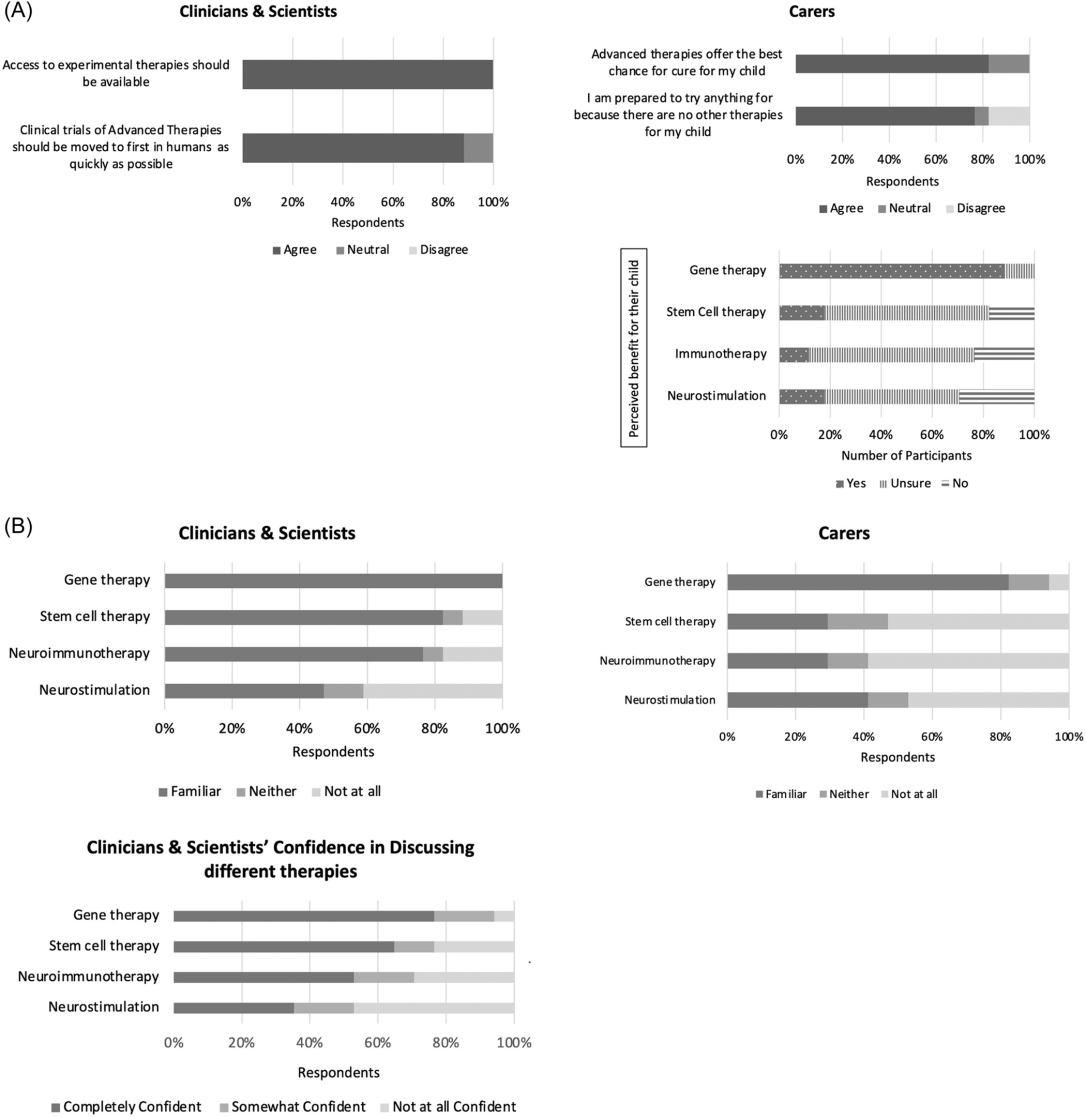
(A) Attitudes of advanced therapies and clinical trials for rare neurological disorders. (B) Understanding of different modalities for rare neurological disorders.

Purposive sampling provided richly textured information and diverse views. Disparities between knowledge and attitudes regarding advanced therapies strongly emerged in both cohorts across qualitative and quantitative findings. All carers and 19/20 (95%) of professionals indicated positive attitudes towards advanced therapies for RNDs; perceived understanding and clinician confidence in discussing advanced therapies were lower and varied between modalities (Figure 1). A total of 13/17 (76%) carers reported a lack of clarity in understanding the process associated with clinical trial involvement, and yet believed that these offered the best therapeutic opportunity for their child.

**Figure 2:**
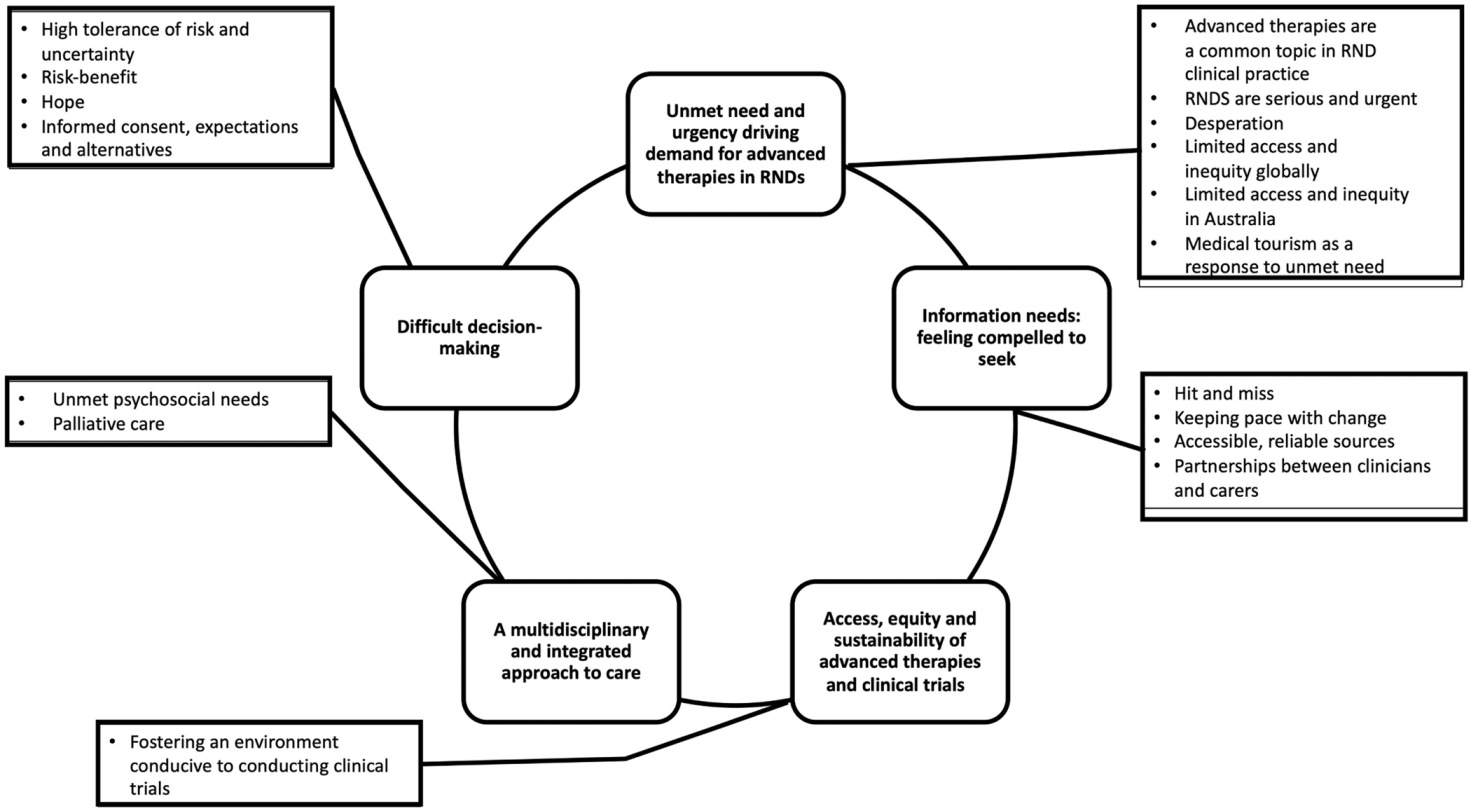
Thematic map summarising qualitative findings. Themes are in bold and located centrally. Subthemes are listed as dot points in the periphery. RND, rare neurological disease.

Five key themes pertaining to advanced therapies for RNDs were identified: (1) great unmet need and complexity; (2) feeling compelled to seek information; (3) access to and equity, and sustainability of advanced therapies and clinical trials; (4) the necessity of multidisciplinary care and support for ongoing health and psychosocial challenges; and (5) difficult decision‐making (Figure 2). Illustrative quotes and survey data for each theme are shown in Table 2.

**Table 2:** Thematic areas and illustrative quotes.

Theme	Subtheme	Illustrative quotes	Participant
1. Unmet need and urgency driving demand for advanced therapies in RNDs	Advanced therapies are a common topic in RND clinical practice	Every time I discuss a rare condition with a family, the next question is … can we fix it?	Clinician/Scientist 5
	RNDs are serious and urgent	you're living with a death sentence … the progression of this disease is described as falling off a cliff, so we knew that every week, every day counted … we didn't have the benefit of time.	Carer 3
		My daughter suffers daily…She has zero quality of life, and if there's some chance that we could improve it – it would be worth the risk.	Carer 10
	Desperation	I'd move heaven and earth anywhere if I thought it was going to help him. I would probably even break the law if I could access it, you know and then that sounds terrible, but you know.	Carer 13
	Limited access and inequity globally	Being in Australia limits our exposure … we do not have the same access to medications as Europe or America.	Carer 16
		We don't have adequate access, we're behind the US and Europe and the UK in terms of priorities in for companies.ind And it's very hard to change that.	Clinician/Scientist 14
	Limited access within Australia	The poor family who, you know, five hours outside Sydney, can't enrol in the research study and they're the ones who may benefit, and the system doesn't allow that to happen.	Clinician/Scientist 2
	Medical tourism as a response to unmet need	I moved to … a country where I didn't speak the language. We found access to this trial and six weeks later, we just got on a plane.	Carer 3
		When we were looking at stem cell therapy … we had to travel so far away … get it done legally.	Carer 13
		Our system drives parents to things that are even riskier.	Clinician/Scientist 16
		A lot of families approach us and talk about medical tourism and take the children overseas for experimental treatments.	Clinician/Scientist 9
2. Information needs: feeling compelled to seek	Hit and miss	I think one of the aspects is that…available information on the Internet is not curated.	Clinician/Scientist 10
		Unless you know where to go or who the experts are, you probably could spend a lot of time trying to find the information that exists.	Clinician/Scientist 13
		We've got this gap opening up paradoxically … we're making more diagnoses—and maybe a handful of those do we find a treatment.	Clinician/Scientist 3
	Keeping pace with change	We cannot keep up with. information about every rare disease… It's up to me to do the research.	Clinician/Scientist 7
		Science and technology and knowledge are always moving forward, and if you If you don't stay up to date with the latest research or new discoveries, etc, you may miss something that could be a great potential.	Carer 1
	Accessible reliable resources	So social media really helps…that's where it travels the fastest.	Carer 12
	Partnership between clinicians and carers	They (carers) could be our biggest advocates.	Clinician/Scientist 16
3. Access, equity and sustainability of Advanced Therapies and clinical trials	Fostering an environment conducive to conducting clinical trials	There is a huge mismatch between patient expectation [around clinical trials] and delivery within the healthcare system.	Clinician/Scientist 7
		You can't make the assumption that every clinical trial is a good clinical trial … we're going to do a bit of gatekeeping.	Clinician/Scientist 3
		We require a lot more trials before they become mainstream, I'm not sure like the process of policies and sign on something getting approved, yeah, it's a bit harder.	Carer 6
		It's such a rare disease, the government excuse is that they really can't afford to fund the treatments.	Carer 19
4. A multidisciplinary and integrated approach to care	Unmet psychosocial needs	There's such a high incidence of depression and anxiety in these parents, so how do we support their psychological and psychosocial needs?	Clinician/Scientist 1
		I know when [my child] was diagnosed I couldn't bear to look at anyone in a wheelchair … there's a lot of grief, …. having the opportunity to have a grief counsellor or a trauma counsellor available is important.	Carer 2
		We literally moved overseas [to access a clinical trial] in six weeks within diagnosis …. we had to act quickly and that also created a huge amount of stress, because you didn't have time to learn a language or put in place support networks or anything.	Carer 3
5. Difficult decision‐making	High tolerance of risk and uncertainty	We understood ultimately that we were really in a dire situation …maybe desperation honestly is the answer.	Carer 15
		I think parents will probably have a feeling of taking greater risk when the alternative is death.	Clinician/Scientist 1
		We just have to try something … even if there's a risk.	Carer 13
		I think most parents with kids with rare new neurological conditions deal with uncertainty every day of their lives… We probably have a higher threshold for them	Carer 16
		Uncertainty all the time with his condition, so it's just another uncertainty, there's nothing guaranteed.	Carer 17
		Risk wasn't just the drug …. the biggest risk is not getting treated.	Carer 3
	Risk–benefit	You're always running the risk of unexpected adverse events by being part of the trial because it's a fairly untested therapy.	Clinician/Scientist 4
	Hope	You're living on adrenaline – it's hope! … if you … can give that child an extra three or four months … you go for it.	Carer 2
		People have really high hopes for these treatments.	Carer 10
	Informed consent, expectations and alternatives	it's a profoundly difficult thing for parents who have a desperately sick child to really objectively evaluate everything that's been laid before them, fully understand it, and make an objective decision.	Clinician/Scientist 3
		I don't think people really … understand what a clinical trial is.	Clinician/Scientist 6
		Informed consent meant almost nothing I would have signed the document in [another language] just to get access to this drug.	Carer 3

Abbreviation: RND, rare neurological disease.

### 3.4 Theme 1: Unmet need, urgency and complexity of RNDs are driving demand for access to emerging advanced therapies

Across all carer and professional perspectives, serious unmet clinical needs were commonly driving interests in, and demands for, emerging advanced therapies. Both cohorts described frequent gaps in effective therapies for RNDs, with 19/20 (95%) carers stating that their child had no disease‐modifying treatments currently available. All carers described their child's debilitating and life‐threatening RND and a concomitant sense urgency and desperation for action.

Where therapies were available to treat RNDs, inequity and obstacles to access emerged as a shared theme for carers and professionals, with 8/20 (40%) carers and 12/20 (60%) professionals expressing frustrations around the differences in therapeutic access to an already limited repertoire of therapeutics both within and outside the Australasian landscape. The role of medical tourism in addressing this gap arose as a subtheme, with 3/20 (15%) carers having experience of international travel for the purpose of accessing experimental treatment, often associated with large financial and personal costs. All professionals empathically recognised the plight and sometimes desperation of families in their therapeutic odyssey. Notably, 8/20 (40%) perceived concerns around the safety, financial burdens and unsubstantiated efficacy of some overseas stem cell and gene therapies. Most (15/20; 75%) professionals highlighted the responsibility and motivation to establish a local ecosystem for enabling the clinical development of advanced therapies for RNDs.

These thematic narratives were consistent with the results of the quantitative surveys, which highlighted the positive expectations and hopes that both cohorts commonly associated with clinical trials (Figure 1). Of the carer cohort, 13/17 (76%) perceived that those clinical trials for advanced therapies presented their child with the best opportunity for treatment access and 12/17 (71%) associated clinical trials with the best opportunity for cure. All professionals “agreed” (11/17; 65%) or “somewhat agreed” (6/17; 35%) that clinical trials for advanced therapies could provide the best treatment options for patients, with 8/17 (47%) endorsing a move to facilitate first‐in‐human studies expeditiously to promote early treatment provision.

### 3.5 Theme 2: Information needs—Compelled to seek

Both cohorts described a dissonance between required knowledge and available information regarding treatment of RNDs. 19/20 (95%) carers described the time‐consuming process of filtering through online information (e.g., Google, social media) and identifying up‐to‐date and trustworthy resources to facilitate decision‐making for their child. Despite their maintained hope for novel therapies, carers expressed a wariness around the variable scientific quality of Internet, social media and patient advocacy information resources. Almost all (19/20; 95%) described challenges in examining and deciphering complex scientific literature, with several (6/20; 30%) stating that they had become well‐versed in scientific theory as a matter of urgent necessity. The quest to connect with global experts arose as an additional strategy of circumventing limited RND resources. Professionals also found it challenging to develop deep, specialised and up‐to‐date knowledge of therapeutic developments pertaining to individual RNDs, considering rapid technological changes, constantly evolving information and time constraints.

While all carers considered their child's clinician as their most trusted source of information, they did not expect them to know everything about RNDs. All carers valued clinician support in interpretation of information. Some (7/20; 35%) professionals described risks associated with information self‐searching amongst carers, cautioning against the potential for unnecessary distress, a lack of control over the timing and content of information and exposure to inaccurate claims and misleading marketing.

Most (9/20; 45%) professionals highlighted the need for collaborative networks of practitioners to facilitate sharing resources and learnings more effectively in the changing therapeutic landscape. Both cohorts unanimously called for appropriately synthesised and curated educational resources around advanced therapies to help guide informed decision‐making for professionals and carers. Quantitative data further characterised an information gap in this space. Less than half of carers (5/17) and clinician scientists (8/17) deemed the information around advanced therapies that was currently available to them as adequate, with only one carer rating the quality as excellent. When ranking preferences for receiving information about advanced therapies, carers ranked their child's medical consultant (10/17; 59%) as their first preference, followed by an in‐person seminar (8/17) and then a webinar (6/17; 47%). Health professionals remained the most trusted sources of health information as noted by 14/17 (%) carers.

### 3.6 Theme 3: Access to, equity and sustainability of advanced therapies and clinical trials

Carers and professionals viewed research and therapeutic development as integral to the clinical care of RNDs, but reported the United States and Europe as world leaders. Based on their experiences, most (18/20; 90%) carers reported feeling lost in the healthcare system while accessing advanced therapies and wanting clarity around treatment pathways, follow‐up care and ongoing interventions that may be required. Professionals and carers placed emphasis on intensifying research and translation efforts as more advanced therapies are ready to be trialled. Professionals proposed actions, including the streamlining of ethics, governance and regulatory processes and establishment of infrastructure to initiate and coordinate trials. Some (3/20; 15%) professionals valued clinical stewardship, ethics committee oversight and governance protocols as necessary in protecting patients. In fact, one professional emphasised the ‘gatekeeping’ role of experts, balancing safety considerations associated with many experimental therapies. Several (7/20; 35%) clinicians/scientists proposed a person‐centred approach in future regulatory evaluations, such as integrating patient perspectives into the assessment of benefits across a continuum of disease severity and manifestations. Almost half (9/20; 45%) of the clinicians/scientists discussed challenges around the high costs of advanced therapies for RNDs, with 17/20 (85%) suggesting a need to ensure sustainable funding and reimbursement models.

### 3.7 Theme 4: The necessity of a multidisciplinary and integrated approach to care

A prominent theme from a professional perspective was the imperative for ongoing multidisciplinary care as the foundation on which health outcomes could be optimised for children accessing advanced therapeutics. In particular, the necessity to proactively offer psychosocial intervention within an advanced therapeutic framework was recognised as an integral part of supporting the child and family unit through what was often a difficult and complex healthcare journey.

Carers' perceptions of unmet needs within the advanced therapy domain aligned with those of healthcare professionals, with emphasis placed on the need for a biopsychosocial model of care to address not only health needs but also the wider psychological ramifications of an RND diagnosis. Carers identified two broad potential areas of psychosocial stress, including embarking on the diagnostic and then therapeutic journey with their child. The grief and emotional distress associated with a diagnosis of RND were linked not only with a prospect of limited survival for some RNDs but also uncertainties surrounding maintaining quality of life for the child. The very search for a ‘cure’ for carers and the complexities of accessing often experimental therapeutics were perceived as a psychologically demanding journey with many highs and lows. From a carer perspective, these complex aspects required integrated and targeted access to psychosocial interventions including ways to establish links with families with similar experiences and early identification of patient advocates within the healthcare journey.

An informal system of family and friends appeared to be the cornerstone of support for many families. In our carer cohort, 12/16 (75%) reported receiving high levels of social support, with ‘significant others’ reported as the most common and highest source of support (14/16; 88%). A majority (10/16; 63%) of carers also reported high levels of social support from friends and family, with a need for family respite to ameliorate the ongoing responsibility of caring for a child with ongoing complex medical needs. While access to palliative care services was noted by some carers to assist in this process of supporting caregivers in their roles, the majority (4/20; 20%) of carers perceived involvement of these healthcare professionals as a last option.

### 3.8 Theme 5: Making difficult decisions

The mechanisms by which families evaluated treatment opportunities and options appeared as a common theme across cohorts. Carers associated this theme with questions around how clinicians could best support and guide decision‐making. Notably, high levels of risk tolerance were associated with levels of unmet (therapeutic) need, correlating with disease severity for carers, despite substantial uncertainties surrounding advanced therapies. Amongst carers, motivations and expectations of accessing advanced therapies varied, ranging from hopes for cure and symptom relief, to supporting research and future discoveries. Some (8/20; 40%) carers were fearful of terrible outcomes associated with advanced therapies, complicating their grief and guilt. Notably, all carers attributed their eagerness for advanced therapies to their perception of the absence of other treatments.

Professionals highlighted their responsibility to manage expectations and guide benefit–risk analyses, with some (9/20; 45%) approaching the topic of advanced therapies with ‘protective pessimism’ (i.e., setting deliberately low therapeutic expectations to guard against disappointment, and raising false hope of unrealistic outcomes, while supporting patient safety). Clinicians raised concerns regarding difficulties in ensuring that carers properly understood the full risks and benefits associated with their decisions. They highlighted challenges of facilitating truly informed choice amidst carers' desperation and uncertainty, the effects of biased information online and the scarcity of well‐developed information and support resources for families.

**Figure 3:**
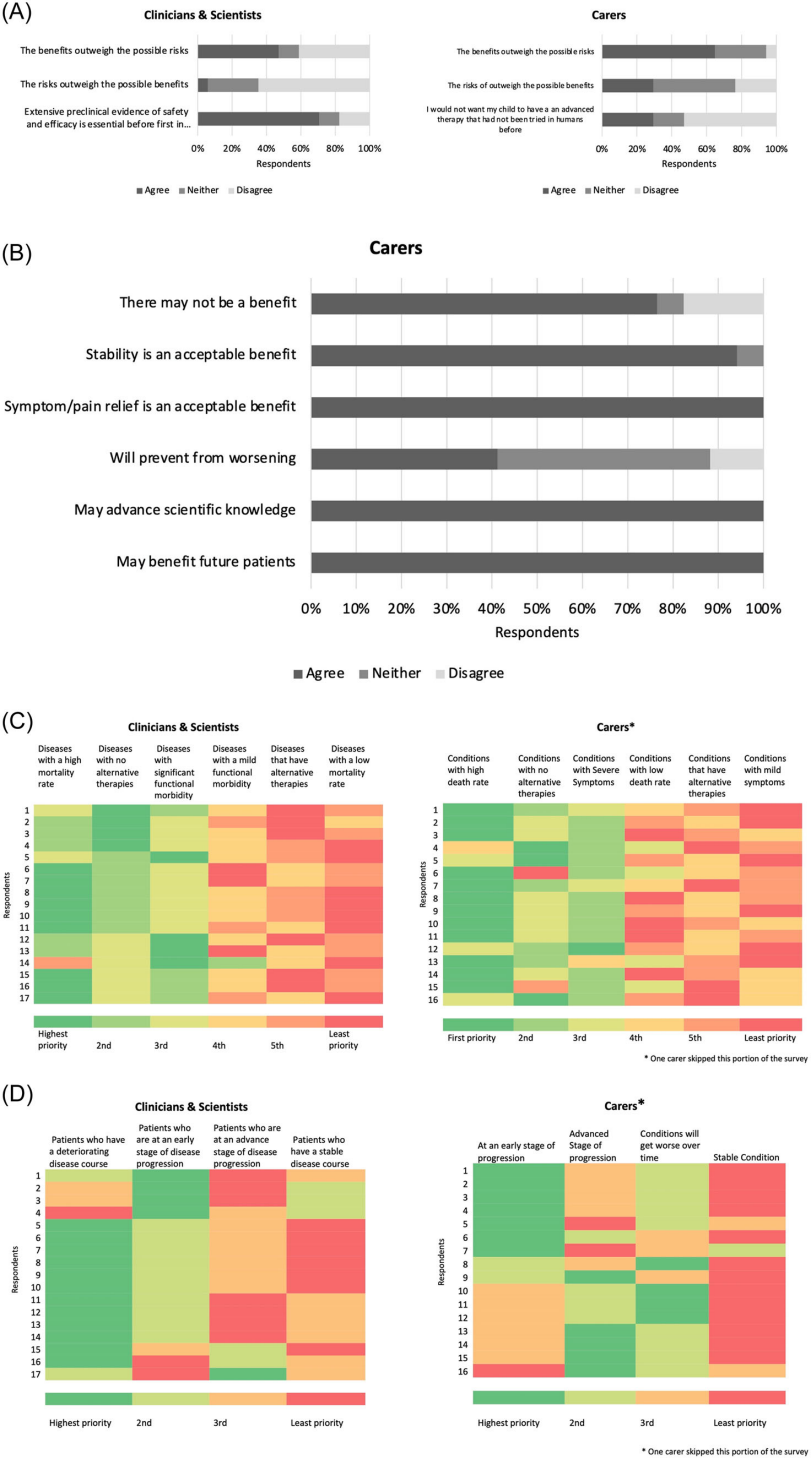
Aspects of difficult decision‐making in the clinical development of advanced therapies. (A) First‐in‐human clinical trials for advanced therapies. (B) Carer perspectives for possible outcomes. (C) Ranking of factors regarding disease prioritisation for the development of novel advanced therapies. (D) Ranking of patient factors regarding prioritisation for the development of novel advanced therapies.

Quantitative survey data were consistent with thematic narratives around difficult decision‐making and prioritisation for therapeutic development. Carers appeared more focused on the benefits of clinical trials and had a higher tolerance of risk; however, professionals' views on clinical trials were more ambivalent. While most (12/16; 75%) carers placed ‘conditions with high death rates’ as their greatest priority when ranking disease prioritisation factors in the development of novel advanced therapies, they lacked consensus around other factors (Figure 3). Such findings were mirrored in qualitative data, which saw carers discuss access in terms of their own child before approaching the question from a broader perspective. In contrast, professionals first approached the topic by recognising its complexity and difficulty, before exploring it via benefit–risk analyses, time course and disease severity, as informed by their field of expertise.

## 4 DISCUSSION

The development of an increasing repertoire of advanced therapies is changing the paradigm of management for childhood‐onset RNDs from a formerly solely supportive model of care to one that has the potential to be more proactive and personalised. This study highlights the contemporary ‘therapeutic odyssey’ that is undertaken by carers and healthcare professionals and the push towards access to clinical trials as a means to start disease‐modifying therapy. Underpinned by the importance of stakeholder engagement and partnerships for successful implementation, the perspectives and lived experiences of stakeholders within the Australian RND community voice a significant disconnect in knowledge surrounding the limitations in eligibility and assessment of impacts and risks of clinical trial enrolment, together with substantial psychosocial burden and information needs to enhance the delivery of care.

While the diagnostic odyssey faced by parents of children with RNDs is well described,^7^ this study's findings attest to the perception that a search for a genetic diagnosis may be better conceptualised as a gateway, through which carers perceive that they may obtain educational and therapeutic service, as well as personal utility including reproductive confidence, connection and explanations. Accordingly, carers and clinicians simultaneously perceive that obtaining a diagnosis is an important but initial stage of an ongoing therapeutic odyssey.^16^, ^17^ The push to develop and access new therapies aligns with the vision as set out by the International Rare Diseases Research Consortium, whose goal is to facilitate the approval of 1000 new therapies for rare diseases, the majority of which would focus on diseases without approved options, by 2027.^18^ However, with over 95% of RNDs currently lacking a recourse to disease modification and many trials being conducted outside of Australia, the discrepancy between stakeholder expectations and realistic access to advanced therapies leaves a gap in which misinformation and uncertainty may predominate.^19^ Our findings indicate that these perceptions may be a significant driver of medical tourism, where the international movement of children to seek advice, diagnosis and treatment often comes at significant financial and psychosocial costs, leaving children with particularly complex needs and low‐efficacy therapeutic options with limited benefits.

These findings have broad ramifications and resonate with the importance of knowledge exchange and partnerships described in an Asia‐Pacific perspective on the facilitators and barriers to implementation of SMA gene therapy.^20^ At the clinician–patient interface, as replicated in prior studies, the importance of a well‐curated, accessible, contemporary and ‘single source of truth’^21^ has been recognised to address information needs in healthcare professionals and carers. While toolkits to improve therapeutic readiness, facilitate knowledge dissemination and inform decision‐making have been built on a disease‐specific basis,^22^ establishing a consensus approach to management and therapeutic decision‐making by concentrating on the shared pathways between heterogenous diseases may enable a broader view of advanced therapies within the context of RNDs across healthcare systems.

Similarly, development of standardised resources that inform the therapeutic journey may enable the unique attributes and complexities of clinical trials in RNDs to be fully understood, empowering carers to partake in informed decision‐making, setting realistic therapeutic expectations, while maintaining hope.^17^ Building on these findings, we are partnering with families to co‐design and co‐produce psychoeducational resources featuring their voices. Moving forward, the development of educational resources for young people is also essential. Studies have shown that actively involving and empowering young people in medical decision‐making and assent processes is valued and associated with lower decisional conflict.^23^, ^24^, ^25^

While professionals and carers were consistent in prioritising clinical trial development for children with RNDs with high mortality, increased functional burden and no alternative therapies, there was paucity of consensus on individual patient factors that should guide therapeutic access. These findings underpin an imperative to address an emergent information gap, assessing individual children based on the risks and benefits of enroling into advanced therapeutic clinical trials while stratifying need based on a prioritisation framework codeveloped by stakeholder consensus. Such guidelines may encompass but are not limited to disease suitability; modality of selecting individual patients; specification of steps needed to enable efficient clinical translation from preclinical research models; standards for clinicians and centres; informed consent processes and identification and assessment of ethical and social issues, such as access, equality and transparency.^26^ While families with high health literacy may proactively seek therapeutic options for their children, social selectivity is a well‐known aspect of clinical trial enrolment.^27^

The international clinical trial and regulatory landscape (including competent authority, ethical and site approvals) is complex, differs extensively between jurisdictions and takes time to operationalise and assess effectiveness, which can impact equitable access to novel therapeutics.^25^ This is in discordance with the push by stakeholders to efficiently access new treatments, as stressed in our present study. A solution may require initiatives to harmonise and coordinate clinical trials within and between borders, streamlining regulatory approvals through ‘master agreements’ while strengthening collaboration amongst clinicians, scientists and researchers across clinical research networks to expedite the translation of advanced therapies into clinical care.

The provision of advanced therapies in healthcare systems requires support beyond medical care, with an imperative to build greater system capacity, support affected individuals and their carers through multidisciplinary team intervention, develop structured care plans and improve psychosocial support for families.^28^ Comparable with previous studies,^29^ 50% of our carer cohort had clinically actionable symptoms of depression and anxiety, with those lacking access to treatment for their child scoring highly for signs and symptoms that characterise psychological disorder. Yet, for carers, the mainstay of psychological support was informal family and friend networks. Our study thus informs the need to recognise multifaceted impacts of RNDs on patient families, proactively survey carers for psychosocial stress and establish early, ongoing mental health and well‐being, financial and disability support to reduce carer burnout and maladaptive coping mechanisms.^30^ Indeed, while previous studies suggest that clinical trial participation may have positive effects on psychosocial functioning and quality of life for children and their parents, they also highlight the importance of providing and maintaining support.^29^

This study is not without its limitations. Our results differ from quantitative and qualitative studies that have characterised poor supports in families affected by rare diseases.^31^, ^32^ Notably, most carers described positive cognitive and behavioural tactics for crisis and distress management, with the majority receiving multiple sources of social supports. Many of our participants had known about their child's genetic diagnosis for several years, allowing them time to establish potentially strong, focussed support networks. Considering the 20% nonresponse rate to the psychosocial surveys, these findings may represent a degree of selection bias. With data saturation and inclusion of several healthcare professionals and carers from various Australian geographical regions, our findings are likely to be relevant nationally. Likewise, the reported experiences from carers in regional/remote regions in our study are similar to the issues detailed in the National Strategic Action Plan for Rare Diseases, namely, significantly limited access to medical treatment, psychological support and specialised multidisciplinary care in RND patients.^19^ To promote integrated and coordinated care inclusive of rare disease neurotherapeutics, further understanding the perspectives of healthcare professionals external to specialised paediatric services is required. Although this study had an Australian focus, the 2021 United Nations resolution *Addressing the challenges of persons living with a rare disease and their families* recognises their shared challenges and emphasises the need for global action to address unmet needs,^33^ supporting the relevance of our findings to the broader international community.

Amidst rapid and promising advanced therapeutic pipelines worldwide, this study provides critical data required to improve models of care for RND so that the health and psychosocial outcomes for children and their families may be optimised. Our findings highlight the need to increase clinical readiness and clinician competency, calling for targeted, evidence‐based and sustainable communication, education and engagement initiatives around RNDs. Globally, these gaps are being addressed, with the exchange of knowledge and recruitment for clinical trials being facilitated by rare diseases reference networks, such as EURORDIS (Home—EURORDIS) and *n* = 1 collaborative (*N* = 1 Collaborative [n1collaborative.org]). Co‐design of educational resources with stakeholders including carers, clinicians and scientists may support a more equitable and family‐centred model of care for children with RND.

## AUTHOR CONTRIBUTIONS

**Christina Q. Nguyen**: Writing—original draft; writing—review and editing; formal analysis. **Didu S. T. Kariyawasam**: Writing—original draft; writing—review and editing; formal analysis; supervision; resources. **Tsz Shun Jason Ngai**: Investigation; writing—review and editing; formal analysis. **James Nguyen**: Investigation; writing—review and editing; formal analysis. **Kristine Alba‐Concepcion**: Investigation; writing—review and editing; visualisation; project administration; resources; data curation; writing—original draft; formal analysis. **Sarah E. Grattan**: Writing—original draft; writing—review and editing; formal analysis. **Elizabeth E. Palmer**: Writing— review and editing; resources. **Kate Hetherington**: Writing—review and editing; resources; formal analysis. **Claire E. Wakefield**: Writing—review and editing; resources. **Russell C. Dale**: Writing—review and editing; resources; investigation. **Sue Woolfenden**: Writing—review and editing; resources; investigation. **Shekeeb Mohammad**: Writing—review and editing; resources; investigation. **Michelle A. Farrar**: Conceptualisation; investigation; funding acquisition; writing—original draft; methodology; validation; visualisation; writing—review and editing; formal analysis; supervision; resources.

## CONFLICTS OF INTEREST STATEMENT

Kristine Alba‐Concepcion receives support from the Ainsworth Foundation. Kate Hetherington receives support from Luminesce Alliance and the Zero Childhood Cancer National Personalised Medicine Program. Claire E. Wakefield receives support from the National Health and Medical Research Council (APP2008300). Sue Woolfenden receives support from the National Health and Medical Research Council (1158954). The remaining authors declare no conflict of interest.

## ETHICS STATEMENT

This study was approved by the Sydney Children's Hospital Network (2020/ETH02748). Written informed consent was obtained from all participants.

## Supporting information

Supporting information.

## Data Availability

The data that support the findings of this study are available from the corresponding author upon reasonable request. Data that underlie the results reported in this article may be available on request, after deidentification. Applicants willing to receive the data should apply between 1 and 12 months after the manuscript has been published and should demonstrate that the proposed use of the data has been approved by an independent review committee identified for this purpose. The data request should be sent to the corresponding author, Michelle A. Farrar.
