# Supplementary material for: ‘High hopes for treatment’: Australian stakeholder perspectives of the clinical translation of advanced neurotherapeutics for rare neurological diseases
Source: Health Expect. 2024 May 6;27(3):e14063. doi: 10.1111/hex.14063 (PMC11074375; doi:10.1111/hex.14063)
Supplement: Supplementary file 1 — Supporting information. [file HEX-27-e14063-s001.docx]

**Supplementary Material 1.**

**Interview guide: Clinician/Scientist**

General information:

- Introduce yourself and the purpose of the research. For families with children who have serious, incurable neurological diseases, advanced and sometimes experimental therapies offer the hope a of treatment for their child’s previously untreatable condition. Families with children who have serious, progressive, and incurable diseases may engage in a state of “therapeutic hope” and pursue experimental therapies at any cost. Clinician’s and scientists have an integral role in supporting patient and family education, managing goals, and guiding therapeutic expectation however may find it difficult to communicate complex scientific aspects, overestimate parents understanding, and unintentional positivity may encourage therapeutic misconception. Providing the balance between being realistic while maintaining hope is also challenging. The purpose of this research is to explore the current uncertainty surrounding advanced therapies, clinicians’ experiences of supporting parents through difficult treatment decisions, managing uncertainty, therapeutic misconception, parental reliance on clinician’s views and the role of palliative care. We will use this information to help inform how we might provide support for clinicians, patients, and parents in the future.
- Ask if participant is happy to have to conversation recorded?
- There are no right or wrong answers, feel free to provide as much or as little information as you like.
- If you feel like you need a break, please feel free to stop at any time.
- Even though we are taping our conversation, nothing you say will be linked back to you personally. The information we gather today will be combined with other interviews and reported as being anonymous.

1. Tell me about your career to date.

- How long have you been working in your current position?
- What qualifications do you have?
- What does your work involve?

1. Tell me about your experiences of advanced therapies for neurodegenerative diseases?

- Have you had any experience with advanced therapies?
- What is your understanding of the benefits of these therapies?
- What is your understanding of the risks of these therapies?
- Have you had any experience of parents seeking information or requesting access to advanced therapies that are not current standard care?
- If yes, talk me through the situation…What was your role? What decision making processes did you go through when responding to the request (for example, what factors did you weigh up, what information did you access or provide, did you make any referrals?),
- Do you think the issues surrounding advanced therapies are similar regardless of the intervention (gene therapy vs stem cell therapy for example) or different?
- Do you feel there is adequate information available for you to be able to be involved in helping families with advanced therapies?
- Do you feel patients have adequate access to experimental therapies in Australia?
- What do you think inhibits access to experimental therapies in Australia? What impact does this lack of access have on families?

1. Tell me about your clinical trial experiences in obtaining informed consent (for experimental treatments)?

- How did you help families understand and navigate the informed consent process?
- Have you ever had concerns that the families didn’t fully understand the purpose or requirements of the trial? How did you manage that?
- Have you had concerns that families didn’t fully understand the risks of an experimental therapy?
- What are the challenges in helping parents understand and process the potential risks of an experimental therapy?
- Based on your experience, what aspects of accessing treatment with advanced therapies do you think families find most challenging to understand? (The aim, the process, the risks, the uncertainty/ limitations in current knowledge?)
- How do you support parents in understanding the benefits of participating in a clinical trial? Do you feel parents fully understand that their child may not benefit directly from a clinical trial?

1. When you think about severe and progressive neurological conditions with limited treatment options, advanced therapies offer hope of effective treatments. However, these treatments come with considerable uncertainty with regards to risks and benefits.

- How do you help families weight up these risks and benefits and manage this uncertainty?
- How do you help families manage and set realistic expectations in the face of uncertainty?
- Do you have any suggestions for how clinicians can support parental hope, without unintentionally encouraging therapeutic misconception? (The tendency to overestimate the benefits of potential therapies)
- Parents often rely on the clinician’s view when making treatment decisions. Has this been your experience?
- As a clinician, how do you feel about this? How do you manage this dynamic?
- Do you have any insight into how to best support parental education and informed decision making?

1. When thinking about advanced therapies, particularly first in human trials where there may be limited pre-clinical evidence, what would you consider to be the minimum amount of pre-clinical evidence necessary for a trial to move to human studies?

- How much uncertainty with regards to potential harms and benefits do you feel is acceptable with these treatments?
- Does this change with the severity of the disease being investigated?
- What types of diseases should be targeted for advanced therapeutic development?
  - Diseases with high mortality and high morbidity?
  - Progressive diseases or stable diseases?
- What individuals do you think are most suitable for selection for first in human clinical trials?
  - Those at early stages of disease progression or later stages of disease progression?
- What family and patient factors need to be considered when offering advanced therapies to families?
  - ability to make informed decisions,
  - families likely or unlikely to comply with trial protocols,
  - the expectations of the family (unrealistic/realistic),
  - their tolerance for risk and uncertainty,
  - families that will “try anything”?
  - Parents understanding/preferences
  - Availability of other options
  - Quality of life

1. What are your thoughts on the role of palliative care when the benefits of advanced therapies are uncertain?

- Do you think the increasing/emerging availability of advanced therapies impacts or will impact the way clinicians/families?

1. What do you think would be helpful for families whose child has just been diagnosed with a serious neurological disorder?

- What do you find helpful/what do you think would be helpful for clinician’s who support families in this situation?

1. Is there anything else you would like to bring up?

**Interview Guide: Carer**

General information:

- Introduce yourself and the purpose of the research. For families with children who have serious, incurable neurological diseases, advanced and sometimes experimental therapies offer the hope a of treatment for their child’s previously untreatable condition. The rapid advances in these therapies pose many challenges for parents including understanding potentially complex scientific aspects of treatment, understanding the risks and benefits of treatment, managing uncertainty, and maintaining hope. The purpose of this research is to explore parents understanding of advanced therapies, their expectations of treatments, and supports and information required to make difficult treatment decisions. We will use this information to help inform how we might provide support for patients and parents in the future.
- Ask if participant is happy to have to conversation recorded?
- There are no right or wrong answers, feel free to provide as much or as little information as you like.
- If you feel like you need a break, please feel free to stop at any time.
- Even though we are taping our conversation, nothing you say will be linked back to you personally. The information we gather today will be combined with other interviews and reported as being anonymous.

1. Tell me about your experiences of your child’s condition?
   - What condition do they have?
   - How old were they when they were diagnosed?
   - Tell me about your child’s current functioning?
   - What do you hope for your child’s future?
2. When you think about treatments for your child’s illness, what comes to mind? (prompts might include lack of treatments, possible new advanced treatments, risk of treatments)

- Tell me about your understanding of gene therapy?
- Are you aware of any gene therapies being developed for your child’s condition?
- If a gene therapy was to become available, what would your expectations be of that therapy for your child?
- What do you understand about stem cell therapy?
- Are you aware of any stem cell therapies being developed for your child’s condition?
- If a stem cell therapy was to become available, what would your expectations be of that therapy?
- Are there any alternative therapies, if so, what are their relative value?

1. What information would you need about the new therapy to help you make a decision about treatment for your child?

- (Possible prompts: risks/benefits, cost, access, processes involved)
- Are there people you would speak to for support or guidance when making a decision about a new therapy?

1. Where would you go to find out information about the therapy?

- What information or supports would you like to assist in making or guiding difficult treatment decisions regarding advanced therapies with uncertain risks and benefits.

1. What is your understanding of the process by which experimental therapies such as gene therapy or stem cell therapy are tested in humans?

- Would you consider your child participating in a clinical trial if it was going to be the first time the treatment had been trialled in humans? Why/Why not?
- What do you consider to be the benefits of participating in first in human clinical trials?
- What do you consider to be the risks of participating in first in human clinical trials.
- Do you feel your child has adequate access to experimental therapies in Australia?
- What do you think prohibits access?
- What do you think would help improve access?
- What would you consider doing to gain access to cutting edge experimental therapies?

After experimental clinical trials are completed, it is typical that patients are followed up over time to see the long term effects of the treatment. What is your view on long term follow up after an experimental intervention?

What is your understanding of the purposes of this follow up?

What are the benefits of a long term follow up?

What are the downsides of a long term follow up?

Should follow up be mandatory?

How long should children be followed for?

The data collected during clinical trials is sometimes available for other scientists to look and use to help develop or improve treatments. In other trials, the data is not made available. What is your view on the sharing of data collected in clinical trials?

Which diseases do you think should be selected for new experimental interventions with substantial uncertainties surrounding the risks and benefits?

Discussion points might include:

Absence or presence of available alternative therapies

High mortality or severe morbidity

Those who are at an early or late stage of their disease course

1. What do you think would be helpful to share with families whose child has just been diagnosed with a serious neurological disorder?
2. What advice would you give yourself 10 years ago now?

- Did your expectations shift over time?
- What processes and timing did you go through in making treatment decisions?
- How do you deal with uncertainties when you provide informed consent to treatments for your child?

Is there anything else you would like to know?

**Supplementary Material 2: Psychosocial tools validation and cut-off scores.**

**DASS-21:** The DASS-21^11^ is the short form of the DASS-42. It is a short self-report tool used to measure symptoms of depression, anxiety, and stress. It is not a diagnostic tool, however, can assist in establishing a diagnosis, screen individuals who may need further evaluation, and monitor outcomes overtime.

It has consistently shown adequate reliability and validity.^11, 34^

A higher score is indicative of an individual experiencing greater symptoms of stress, anxiety, or depression. An individual receives a score for the 3 subscales (stress, anxiety, and depression). These scores are categorised into the following severity ranges, with the score required for each range detailed below.

|  | Stress | Anxiety | Depression |
| --- | --- | --- | --- |
| Normal | 0-7 | 0-3 | 0-4 |
| Mild | 8-9 | 8-9 | 5-6 |
| Moderate | 10-12 | 10-12 | 7-10 |
| Severe | 13-16 | 13-16 | 11-13 |
| Extremely severe | 17+ | 17+ | 14+ |

**The COPE Inventory:** The COPE Inventory^12^ is a validated, 60 item self-report tool that reports theoretical assumptions of coping styles. On a 4-point Likert scale (1 = I usually do not do this at all; 2, I usually do this a little bit; 3= I usually do this a medium amount; 4 = I usually do this a lot), respondents are asked to select the frequency at which they engage in various coping styles that are categorised into 15 groups. These coping styles include acceptance, active coping, behavioural disengagement, denial, seeking emotional support, humour, seeking instrumental support, mental disengagement, planning, positive reinterpretation and growth, religion, restraint, substance use, and suppression of competing activities. The highest score that can be achieved for each coping style is 16, and the lowest score is 4. The COPE Inventory has good psychometric properties and has shown adequate validity and reliability.^12^

**The Multidimensional Scale of Social Support (MSPSS): The** MSPSS^13,35^ is a 12-item self-report questionnaire to measure perceived adequacy of social support from three sources: Family, friends, and significant others. 4 questions are allocated to each subscale. A 7-point Likert scale is provided for respondents to select the most appropriate answer against all questions (1=Very strongly disagree; 2=strongly disagree; 3=disagree;4=neither agree nor disagree; 5=agree; 6= strongly agree; 7= very strongly agree). A higher score is indicative of greater perceived social support. The tool has shown adequate validity and reliability across several studies, including in populations of parents and carers of children with a neurological condition.^36^

**Supplementary Material 3: Participant Characteristics.**

| Participant ID | Gender | Age Range | Profession | Location | Child’s Condition | Has Accessed Advanced Therapies? Y/N |
| --- | --- | --- | --- | --- | --- | --- |
| Clinicians/Scientists | | | | | | |
| Clinician  /Scientist 1 | F | 61-70 | Paediatric Neurologist | NSW | n/a | n/a |
| Clinician  /Scientist 2 | M | 61-70 | Clinical Geneticist | NSW | n/a | n/a |
| Clinician  /Scientist 3 | M | 61-70 | Geneticist and scientist | NSW | n/a | n/a |
| Clinician  /Scientist 4 | F | 41-50 | Paediatric Neurologist | NSW | n/a | n/a |
| Clinician  /Scientist 5 | F | 41-50 | Paediatric Neurologist | NSW | n/a | n/a |
| Clinician  /Scientist 6 | F | 31-40 | Genetic Counsellor | NSW | n/a | n/a |
| Clinician  /Scientist 7 | M | 51-60 | Metabolic Physician | NSW | n/a | n/a |
| Clinician  /Scientist 8 | M | 51-60 | Paediatric Neurologist and scientist | NSW | n/a | n/a |
| Clinician  /Scientist 9 | F | 41-50 | Laboratory scientist | NSW | n/a | n/a |
| Clinician/Scientist 10 | M | 41-50 | Paediatric Neurologist | NSW | n/a | n/a |
| Clinician  /Scientist 11 | M | 51-60 | Paediatric Neurologist and scientist | VIC | n/a | n/a |
| Clinician  /Scientist 12 | F | 51-60 | Clinical Geneticist | NSW | n/a | n/a |
| Clinician  /Scientist 13 | F | 41-50 | Allied health scientist | NSW | n/a | n/a |
| Clinician  /Scientist 14 | M | 41-50 | Paediatric Neurologist | VIC | n/a | n/a |
| Clinician  /Scientist 15 | F | 51-60 | Paediatric Neurologist | QLD | n/a | n/a |
| Clinician  /Scientist 16 | F | 51-60 | Neonatologist and scientist | NSW | n/a | n/a |
| Clinician  /Scientist 17 | F | 41-50 | Genetic Counsellor | NSW | n/a | n/a |
| Clinician  /Scientist 18 | F | 31-40 | Genetic Counsellor | NSW | n/a | n/a |
| Clinician  /Scientist 19 | M | 41-50 | Laboratory scientist | NSW | n/a | n/a |
| Clinician 20 | F | 51-60 | Paediatric Neurologist | NSW | n/a | n/a |
| Primary Carers | | | | | | |
| Carer 1 | M | 41 -50 | n/a | NSW | Neurodevelopmental | Y |
| Carer 2 | F | 41 - 50 | n/a | NSW | Neuromuscular | N |
| Carer 3 | F | 41 - 50 | n/a | NSW | Neurodegenerative | Y |
| Carer 4 | F | 61-70 | n/a | NT | Neurodegenerative | N |
| Carer 5 | F | 31-40 | n/a | NSW | Neuromuscular | Y |
| Carer 6 | F | 41 - 50 | n/a | NSW | Neurodegenerative | Y |
| Carer 7 | F | 41 - 50 | n/a | NSW | Neuromuscular | Y |
| Carer 8 | F | 51- 60 | n/a | NSW | Neurodevelopmental | Y |
| Carer 9 | F | 31- 40 | n/a | NSW | Neurodevelopmental | Y |
| Carer 10 | F | 31 - 40 | n/a | NSW | Neurodevelopmental | N |
| Carer 11 | F | 41 -50 | n/a | NSW | Movement Disorder | N |
| Carer 12 | F | 41 -50 | n/a | NSW | Neurodegenerative | N |
| Carer 13 | F | 41 -50 | n/a | NSW | Neurodevelopmental | Y |
| Carer 14 | F | 31 - 40 | n/a | NSW | Neurodevelopmental | N |
| Carer 15 | M | 41 - 50 | n/a | USA | Neurodegenerative | N |
| Carer 16 | F | 31 - 40 | n/a | NSW | Neurodevelopmental | N |
| Carer 17 | F | 41 - 50 | n/a | NSW | Neuromuscular | N |
| Carer 18 | F | 41 - 50 | n/a | NSW | Neurodevelopmental | Y |
| Carer 19 | M | 41 - 54 | n/a | NSW | Neuromuscular | Y |
| Carer 20 | F | 51 - 60 | n/a | NSW | Movement Disorder | N |
